# Factors promoting and inhibiting sustained impact of a mental health task-shifting program for HIV providers in Ethiopia

**DOI:** 10.1017/gmh.2017.21

**Published:** 2017-12-04

**Authors:** D. Jerene, M. Biru, A. Teklu, T. Rehman, A. Ruff, L. Wissow

**Affiliations:** 1 Management Sciences for Health, Addis Ababa, Ethiopia; 2 Department of Heath Science, Faculty of Medicine, Lund University, Lund, Sweden; 3 MERQ Consultancy Services PLC, Addis Ababa, Ethiopia; 4 Yale School of Medicine, New Haven, CT, USA; 5 Department of International Health, Johns Hopkins School of Public Health, Baltimore, MD, USA; 6 Division of Child and Adolescent Psychiatry, Johns Hopkins School of Medicine, Baltimore, MD, USA

**Keywords:** Mental health, sustainability, task-shifting

## Abstract

**Background.:**

Task-shifting mental health into general medical care requires more than brief provider training. Generalists need long-term support to master new skills and changes to work context are required to sustain change in the face of competing priorities. We examined program and context factors promoting sustainability of a mental health task-shifting training for hospital-based HIV providers in Ethiopia.

**Methods.:**

Convergent mixed-methods quasi-experimental study. Sustained impact was measured by trained/not-trained provider differences in case detection and management 16 months following the end of formal support. Factors related to sustainability were examined through interviews with trained providers.

**Results.:**

Extent of sustained impact: Trained providers demonstrated modest but better agreement with standardized screeners (greater sensitivity with similar specificity). They were more likely to request that patients with mental health problems return to see them *v.* making a referral. Factors promoting sustainability (reported in semi-structured interviews): provider belief that the treatments they had learned were effective. New interactions with on-site mental health staff were a source of ongoing learning and encouragement. Factors diminishing sustainability: providers feelings of isolation when mental health partners left for work elsewhere, failure to incorporate mental health indicators into administrative data, to re-stock staff education materials, and to build formal mechanisms for generalist-mental health staff interaction.

**Conclusions.:**

An intervention seen as feasible and effective, and promotion of relationships across professional lines, helped generalists sustain new skills. Failure to address key system context issues made use of the skills unsustainable as external supports ended.

## Introduction

Providing care through general medical providers and community health workers is a central strategy for expanding access to mental health services (Patel *et al.* 2013). Generalists express interest in addressing mental health problems, but worry about taking on new responsibilities without long-term educational and referral support (Petersen *et al.* 2011; Mendenhall *et al.* 2014; Stein *et al.* 2016; Straus & Sarvet, 2014). Sustainability is thus a critical element of programs that seek to build generalists’ mental health care capacity.

The JHU-TSEHAI Program for Mental Health Problems in HIV Care (Legesse *et al.* 2011; Wissow *et al.* 2014) was part of the US President's Emergency Plan for AIDS Relief (PEPFAR) in Ethiopia. Program components included attention to context (support from federal, regional, and local health authorities, supplies of required medications, designing content in collaboration with Ethiopian experts), brief mental health training for generalist HIV providers (covering front-line approaches to common mental disorders across the lifespan as well as epilepsy, psychosis, and dementia), and short-term support in the form of printed reference materials and follow-up site visits over a period of a few months. Co- and cross-training of HIV providers and mental health staff were included to build a common vocabulary, shared sense of mission and long-term, self-sustaining collaborations (Benzer *et al.* 2012; Wilkes & Stopforth, 2015). The program's reference guide for clinicians and facilitator guide are available at www.jhu.edu/pedmentalhealth.

The program was originally implemented in seven clinics in four regions of Ethiopia over a period of about 3 years (November 2010–February 2013). In an informal, mid-roll-out evaluation, conducted by interviewing trainees at four sites where training had already taken place, HIV providers reported that they were more likely than pre-training to listen to and counsel patients with low mood or anxiety. New linkages were developing with on-site mental health staff, some of whom had over time been assigned to non-mental health roles but now were allowed to return to mental health work. Staff turnover and lack of ways to easily document mental health-related encounters were reported as problems; these were addressed by creating new brief clinical reference materials and a simplified mental health registry (Legesse *et al.* 2011).

In June 2014, about 16 months after the last training, we returned to two of the sites to look for evidence of the program's ongoing impact. We used Stirman *et al*. (2012) model to explore factors potentially promoting ongoing impact (Table 1). Stirman *et al.*, identify four main influences on program sustainability: characteristics of a program's core intervention (including effectiveness and fit with its context), characteristics of the program's context (aspects of the setting, participants, and leadership), the setting's capacity to carry out the program (funding, workforce), and processes available at the setting (including mechanisms for ongoing evaluation and feedback, the creation of working relationships among stakeholders) (Table 1). We use this framework to understand persistence of the program. We were particularly interested in whether the attempt to promote local collaborative relationships had been able to sustain the HIV providers’ mental health work after external support for the program had ended.

**Table 1.** Characteristics of the HIV-mental health integration intervention related to sustainability

Influences on sustainability^a^	Promoted sustainability	Hindered sustainability
Intervention characteristics		
Flexibility/fit	Content fit will with patient needs and built on prior training in HIV counseling	Inability to address social determinants of mental health problems could be frustrating to staff
Effectiveness or benefit	Trained staff felt material was effective, benefited patients	
Ability to maintain fidelity	Promotion of inter-professional collaboration built knowledge; charts and ‘pocket guides’ useful	Lack of booster sessions to support and create new collaborations and maintain attention to program
Context		
System/policy change	Government interest in integrated care as vehicle for expanded access to mental health services	Though endorsed by leadership no long-term champions emerged at local level
Setting characteristics (structure, policies)		High volume patient care, lack of privacy; policies requiring nurses to rotate services; fixed national medical record not allowing mental health care to be documented
Capacity		
Workforce	Elevated status of mental health nurses and/or returned them to mental health roles	Continued turnover in HIV staff; mental health not part of on-boarding of new HIV staff
Processes		
Building relationships	Increased informal bi-directional consulting between HIV and mental health staff	Lack of formal joint activities such as rounds or team meetings
Mechanisms for evaluation and feedback		Absence of screening and documentation requirements that could have facilitated evaluation

^a^Categories from Stirman *et al.* 2012, Table 2.

## Methods

### Study design

This study used a mixed-methods quasi-experimental design (Campbell & Stanley, 1963; Fetters *et al.* 2013). Data are available only at a ‘post’ intervention time point and contrast care given by HIV providers who did and did not receive the program training.

### Measuring impact

We first assessed whether there had been sustained impact, using the framework provided by Shediac-Rizkallah & Bone (1998), which looks for evidence of ongoing benefits, activities, and capacity. The main quantitative measure of sustained impact is difference in provider recognition of patients with possible mental health problems, comparing trained and untrained HIV providers. We hypothesized that trained providers would better identify patients with mental health problems (greater sensitivity and specificity) compared with untrained providers. We recruited patients of trained and untrained providers, administered age-appropriate standardized screening tools, and, without revealing screening results, asked providers to indicate if they believed the patient they were seeing had a mental health problem requiring intervention. If so, providers were asked what type of problem they believed the patient had (thought problems, depression, anxiety/trauma, epilepsy, child development/conduct, substance use, difficulty living with HIV – categories used in the training) and what treatment or follow-up plans they provided, if any. Subsequently, screening results were unmasked and patients offered further assessment if results suggested that it was needed.

### Sites

The evaluation took place in two of the program's original seven sites, those most accessible (the others were located hours to days travel outside the capital) and with the highest volume of HIV care (together they served 71% of all patients across the sites). At both evaluation sites, HIV clinics provide diagnostic and long-term treatment services. Both have ambulatory mental health services on their campuses; at Site 1 these are provided by nurses and at Site 2 by nurses and psychiatrists. Neither site has inpatient mental health care. Site 1 had experienced turn-over in HIV staff and at the time of the evaluation had both trained and untrained providers; at Site 2 all active HIV staff had received mental health training.

### Providers

We enrolled all active HIV providers at the two sites (*n* = 33, 21 from Site 1 and 12 from Site 2). Nine were physicians, 22 were nurses, and two were health officers; 19 had received the program training and 14 had not. Not all of the enrolled providers ultimately participated in the study: participation depended on: (a) if one or more of their patients made a visit during the quantitative part of the study, or (b) if they were selected at random for an interview as part of the qualitative part of the study.

### Patients

Patients were eligible if they were coming to see one of the enrolled providers and were age 6 or older. They or their parents were approached and asked for written consent by a member of the site clinical team (not participating in the study). Recruiting took place over 9 days, involving consecutive patients of any enrolled providers working on those days.

### Data collection

If patients agreed to participate those 16 and older were administered the Amharic versions of the Kessler 10, a brief assessment for common mental disorders (Tesfaye *et al.* 2009) and the AUDIT, a brief screen for harmful alcohol use (Soboka *et al.* 2014). In one validation study against a structured clinical interview for depression in Ethiopia, the Kessler 10 had an area under the ROC curve of 0.91 and a Chronbach's *α* of 0.90 (Tesfaye *et al.* 2009). The Ethiopian version of the AUDIT has been modified to reflect the alcohol content of traditional beverages and demonstrated concurrent validity against reports of risk factors for drinking (Soboka *et al.* 2014). For those under 16, parents completed an Amharic translation of the Strengths and Difficulties Questionnaire (Goodman, 1999; Goodman *et al.* 2000; www.sdqinfo.com). While the SDQ has been used in studies in Ethiopia, it has not been formally validated. In addition, we extracted from patients’ medical records information about their general health and HIV treatment status.

For the qualitative portion of the study, interviews were conducted in Amharic by a member of the study team (MB) who had not been part of the JHU-TSEHAI program development or delivery. The interview guide (for English translation see online Supplementary Appendix) elicited provider, patient, and contextual factors that, in the eyes of providers, facilitated or hindered delivery of mental health care in the HIV clinics. For trained providers, the interview also asked about aspects of the JHU-TSEHAI training perceived as helpful and the extent to which activities promoted by the training were still taking place. The guide was adapted from the evaluation of a US task-shifting program involving community-based providers of children's health care (Gadomski *et al.* 2014).

### Sample size

For the quantitative portion of the study, we aimed to enroll approximately 150 patients at Site 1 to determine a difference between moderate and low levels of agreement with screening instruments by trained *v.* untrained providers, assuming *α* = 0.05, power = 0.8. Calculations used the ‘R’ statistical program's ‘N.cohen.kappa’ function.

A sample of 12 enrolled providers (six from each site) was randomly selected for the interviews described above. The sample was drawn by research associates prior to any quantitative data analysis. Because this sample did not yield data saturation for untrained providers, we recruited four additional providers from Site 1 who had not been trained.

### Data analysis

Kappa and other bi-variate statistics were computed to assess agreement between provider identification of mental health problems and screening results. Stata procedure xtmelogit was used for final summary analyses to account for clustering of patient-level observations within providers.

Qualitative analysis used a ‘constant comparison’ approach feeding back thoughts about interview content and making adjustments to the interview (Glaser & Strauss, 1967). Interviews were conducted in Amharic, recorded, and translated into English by MB. LW read and re-read the English translations line by line and developed a codebook based on the interview guide. TR then re-read the interviews and made suggestions for merging some codes and creating additional categories. TR and LW then coded four interviews and met to resolve differences; LW then coded the remaining interviews. Coding and abstraction used the TAMS Analyzer (version 4.0; University of Washington-Tacoma).

## Results

### Recruited providers

Among the providers of recruited patients in the quantitative portion of the study (*n* = 11) four were physicians and seven were nurses. Four were trained (all nurses) and seven were not. Among providers in the qualitative portion of the study (*n* = 16), four were physicians and 12 were nurses. Eleven had been trained (two physicians and nine nurses) and five had not been trained (two physicians and three nurses).

### Recruited patients and screening results

At Site 1, 148 patients were approached and agreed (directly or via a parent) to participate – none of those approached declined. They ranged in age from 7 to 75 years (mean 37, s.d. = 12); 103 were female and 45 male. All but two were described as having a functional status allowing them to work or attend school. Patients’ most recent CD4 cell counts averaged 506 (median 479, range 9–2029); 11 patients (7%) had CD4 counts <200.

Across all three screening instruments, 44 of the 148 patients (30%) were rated as having a mental health problem (positive on any of the three). The positive included three of 15 children (20%) on the SDQ, 37 of 133 adults (28%) on the Kessler 10, and 8 (6%) on the AUDIT.

Patients of trained and not-trained providers did not differ significantly by gender (67% female *v.* 71%) or positive screening results (33% *v.* 27%), but patients of trained providers were younger (mean age 32 *v.* 39 years, *p* < 0.001).

### Evidence for sustained program impact

Trained providers’ agreement with screening results was modest but better than among untrained providers (*κ* = 0.40, *p* < 0.001 *v*. 0.24, *p* = 0.01). Compared with the screening results, trained providers’ sensitivity was 89% and specificity 58%; untrained providers’ sensitivity and specificity were 64% and 65%, respectively. Adjusted for patient clustering among providers, the odds that trained providers would correctly identify a screen-positive patient was 7.3 times greater than the odds for non-trained providers (95% confidence limits 1.0–54.4). The odds that trained providers would incorrectly label as positive a screen-negative patient was 1.2 times greater than non-trained providers, a difference that was not statistically significant (95% confidence limits 0.55–2.9).

This difference in ability to identify patients with mental health problems was reflected in providers’ interviews. Trained providers described how they had begun to ask patients about mental health problems, while those who had not been trained continued to feel that there was insufficient time to respond to any but the most serious and obvious problems (Table 2).

**Table 2.** Evidence for sustained impact: contrasting excerpts from interviews with trained v. not-trained providers

	Trained provider	Not-trained provider
Attitudes toward incorporating mental health in HIV care	I feel that we have to work on mental health problems aggressively. Continuous updates (trainings, guidelines, brochures) on the area will give us more confidence so as to opt to manage such cases rather than ignore them. With sufficient training and support I will be more than happy to work on mental health problems. – 207	I always put myself in the shoes of others: I really feel and share the challenge and pain of my clients. If I had adequate knowledge in the area with good training I would have been supporting many clients with this problem. – 503
Attitudes toward detecting mental health problems	The training has helped me to be more conscious of mental health problems, which I used to overlook before. Prior to the training, I had no understanding as to how to approach patients with such problems because I didn't have sufficient knowledge on the area. Moreover, I used to ignore such cases because I felt it was not a serious problem at all. – 206	Unfortunately due to our facility high client load I don't have much time to deal with patients with mental health problems. Only very few things I try to discuss and provide them (with help). I usually send them to the psychiatric clinic even though I know that the service might be substandard there due to lack of adequate trained manpower. – 501
Types of problems noted	Before the training, I was not that conscious about the need for mental health assessments. My approach was very ordinary like ‘How are you? Why do you be stressed? Be courageous …’ and was not professional. After the training, however, I do not see (take) anxiety for granted. – 212	Both of them [patients with mental health problems] were presenting with almost similar behaviors – they were disturbing others, aggressive, and unstable. They were not willing to listen to others. – 502 I observed some clients with the symptoms of irritability, getting easily anger, unwilling/not happy to continue with their treatment. Overall, the symptoms indicated that these clients were not mentally stable. – 500
Decisions to refer	We see many stressful clients – some of such cases may be rehabilitated by intensive counseling and some not. Based on the level of severity of the problems we either handle them here providing allowable treatments (like amitriptyline) or we refer to the psychiatry unit. – 304	We are extremely busy and can't spend time with emotionally disturbed clients. No single organization can address all their needs. We should be aware of their problems, not ignore them but it is a joint effort (with psychiatry). – 305 He used to visit our clinic with no scheduled appointment… Finally I referred him to psychiatry clinic and the psychiatry clinic again referred him to [the psychiatric hospital]. But still he is with the same problem after 6 months. – 500

When providers considered that a patient had a mental health problem, the distribution of provisional diagnoses assigned by trained and untrained providers was similar with two exceptions (overall χ^2^ *p* < 0.05). Untrained providers identified 16% of those they believed had problems as having a ‘thought problem,’ while none of the trained providers gave a patient this label, and trained providers identified 23% of those with problems as having anxiety compared with 10% among those identified by untrained providers. These differences in interpretation of patients’ symptoms and behavior were reflected in the interview responses. Trained providers talked about asking patients about emotional concerns, and how they had become more aware of patients’ affective and social problems. Untrained providers emphasized that their patients exhibited irritability and aggression or were unwilling to take medication. As one trained provider said, ‘before the training I was not that conscious about the need for mental health assessments. My approach was… “Be courageous” and was not professional. After the training I do not take anxiety for granted.’

Both trained and not-trained providers offered some form of treatment (discussion within the visit, medication, or referral) to patients they believed had a mental health problem (74% *v.* 68%, *p* = 0.53). There were no significant differences in the proportion of identified patients given a medication (17% *v.* 8%), receiving some form of counseling during their visit (40% *v.* 44%) or given follow-up of some kind (43% *v.* 28%). However, there were differences in what trained providers did within these categories. In arranging follow-up visits, not-trained providers referred all of the patients they identified to a mental health specialist, while trained providers asked half (8/15, *p* < 0.01) to return to see them in the HIV clinic. For their in-office discussions, only trained providers used psychoeducation (7/12. *p* < 0.01) *v.* more general ‘counseling.’ These differences were reflected in the interview responses. Not-trained providers mentioned immediately referring when possible, while trained providers described a mix of responses depending on the patients’ level of severity, including providing some initial treatment and scheduling a follow-up visit before deciding on the need for a referral.

Trained providers’ comments suggested that participation had changed their attitudes toward the appropriateness of detecting mental health problems and intervening in the HIV clinic. While both trained and untrained providers saw mental health as important to HIV care, trained providers said they were now willing to take it on while not-trained providers still saw it as something for which they were not prepared. As one trained provider put it, ‘The training has helped me to be more conscious of mental health problems, which I used to overlook before.’

### Influences on sustainability

Providers’ interview responses suggested that factors related to all four key domains of sustainability played a role in the evolution of mental health care after program support ended. The main findings are summarized in Table 1 and selected quotations from informants are shown in Table 3.

**Table 3.** Influences on sustainability: excerpts from interviews with trained providers

	Favors sustainability	Hinders sustainability
Program factors		
Perceived effectiveness	I remember my client who started to hallucinate after he took ART for 5 months. At the time I had already taken the training. I treated him and helped him recover from the problem without a need to refer him to a psychiatrist. He totally recovered after 15 days of treatment and follow up – and was back to his business. – 201	We usually observe mental health problems in mothers who are economically deprived, who divorced/betrayed by their husbands, who have several children, etc. It is common that husbands abandon such mothers when they learn of their HIV status – and that leads them to stress that again leads to mental health problems. We usually provide strong counseling for such clients but sometimes we end up fruitless. – 302.
Perceived fit	Definitely [training] helped me to be conscious of mental health problems, easily identify such cases, and link with our mental health clinic when need be… Without the training I wouldn't have managed such clients as intended. – 303	So not only health facilities but also other civil society organizations, financial institutions, the media, and other stakeholders should work together to mitigate the impact (of chronic illnesses such as HIV, TB, and leprosy). – 305
Promoting new partnerships	[The training] enhanced the level of communication and collaboration between my team and the psychiatry unit. Currently the two units have a very good understanding and (more) synergy than ever before. – 207	I consult my supervisor and colleagues when I face challenges. It is a teamwork and no one can handle all cases on its own. Previously we also had a regular professional counseling and discussion sessions with mental health specialists from OPD. It is no longer happening now after the lead mental health specialist left the facility. – 212
Contextual factors		
Burdens and competing demands		I am highly interested to support such clients as far as my work environment allows, but it takes time. 301
Physical space		For the sake of privacy we sometimes abstain ourselves from asking some important questions. 302
Support from management		There are inconsistencies in service provision, which is majorly related to loose commitment of staff. If we are committed, we have the knowledge and so we can deliver. – 301
Inclusion in new staff orientation		You may get surprised. I only know very recently even about the presence of one psychiatrist at the facility. I don't know really whether there is any conditions which conducive for mental health management in the facility. – 502
Quality of partnerships	This week I faced a controversial issue regarding a patient that was referred to me from psychiatry unit. I discussed with doctors in my unit and diagnosed him for toxoplasmosis. Currently we are waiting for the result. In case he is diagnosed negative for toxoplasmosis we will send him back to the psychiatry unit for further diagnosis. 207	Not at all [ever provided MH care to ART patient] due to several reasons. One thing I am a new employee and the other is I expect I am not supposed to work in the clinic for longer duration due to the need of rotation to other units according to the facility regulation. I may rotate to emergency clinic and wards too. – 501

#### Intervention characteristics that promoted sustainability

Several characteristics of the intervention were cited as promoting both its initial implementation and its sustainability. These included its perceived effectiveness, the fit of the intervention with prior training in HIV counseling, and the successful building of new on-site interprofessional relationships.

##### Perceived effectiveness

Trained providers felt they now could help patients in ways that they had not previously been capable of. This was a benefit to both patients and staff. One trained provider noted that he was now less likely to ‘burn out of repeated cases because I always know they can be treated and recover.’ Providers’ sense of effectiveness came in part from patient feedback. As one provider said, ‘If my clients thank me simply because I gave them amitriptyline and they were relieved temporarily, how come they can't be even happier if I give them a full-fledged mental health treatment?’

##### Perceived fit

The training appeared to fit well with providers’ existing capabilities, the kinds of mental health problems that patients frequently experienced, and with providers’ prior training experiences in counseling. The training covered problems that the providers had felt were both common and frustrating, and in that way met a need.

The intervention also seemed to fit well with patient preferences, as perceived by the providers. They reported that their patients liked the idea of ‘not having to tell their story again’ (as they would have had they been referred) and preferred to receive treatment at a location not associated with the stigma associated with mental health.

##### Promotion of new inter-professional collaboration

Providers noted that the training had increased their working relationships with the mental health providers at their sites. Particularly important was having been trained by mental health clinicians who would later be available for ongoing consultation, as well as a sense that even clinic staff not directly involved in the patients’ primary medical care (for example, pharmacists) had been trained and shared common goals.

#### Intervention characteristics that hindered sustainability

##### Limitations of fit with patient population

Providers pointed out that many of their patients had difficulty returning to see them for follow-up, largely because the patients had only come to the metropolitan area temporarily to initiate HIV care. In addition, patients’ mood and anxiety were seen as being heavily influenced by social circumstances that were beyond the help providers could offer, despite attempts to link with community-based services. These limitations detracted from a sense of the training's effectiveness. One provider noted that many patients, particularly mothers of HIV+ children, had serious economic problems and were commonly abandoned by their husbands. ‘We usually provide strong counseling for such clients,’ she said, ‘but sometimes we end up fruitless.’

##### Continued dependence on outside support for refresher training and materials

Despite the initial success of building local collaborations, as key mental health consultants left for other positions, the HIV providers lost their source of ongoing mental health support. Materials distributed at the time of the training, such as posters illustrating diagnostic algorithms, were taken down or damaged and not replaced. ‘Refreshers’ and the provision of updated materials were seen as important not just for the information they might provide but also as a sign that the topic remained a priority. Booster sessions attended jointly by both HIV and mental health providers might have been able to develop new local collaborations as staff turned over.

#### Contextual factors that promoted sustainability

The program benefited from trends in the larger world of mental health and non-communicable disease care in low and middle income countries. The World Health Organization's Mental Health GAP program (mhGAP) was being developed simultaneously, promoting mental-health related support and discussion within the Ethiopian Ministry of Health (Anonymous, 2013). JHU-TSEHAI staffs were able to contribute to editing of a national mental health strategic plan, which also helped cement relationships at the ministry level (Federal Democratic Republic of Ethiopia, 2012). The TSEHAI program's training manual had an introduction signed by the Minister of Health and featured the ministry logo on its cover. Ministry support, in turn, promoted support from regional health authorities and hospital administrators; the latter provided key support related to sustainability in the form of space (at Site 1, a mental health office that had been turned over to another program was returned to mental health care) or manpower (restoring the roles of mental health nurses who had been re-assigned to general medical rather than mental health duties).

#### Contextual factors that hindered sustainability

Several aspects of context hindered sustainability, including the volume and pace of patient care, aspects of the physical setting in which care was delivered, and a perception that higher management at the intervention sites was not committed to mental health care.

##### Burden of patient volume

Providers reported that they felt overloaded in both patient volume and the number of tasks to accomplish with each patient. Despite interest in mental health care, there was always the ‘push back’ of other demands.

*The physical setting* of the sites also made it difficult to consistently raise mental health issues. Multiple visits might be occurring in the same large office equipped with several desks and examining tables; HIV patients and those with other primary problems might be seen in the same clinic within earshot of each other. As one provider noted, ‘For the sake of privacy we sometimes abstain from asking some important questions.’ This had been a challenge to initial implementation and it persisted as a force counteracting efforts to sustain mental health care.

##### Lack of perceived support by upper management

As outside support ended, providers perceived a lack of ongoing interest in the mental health care on the part of the administration and outside supporters. While individual providers had become interested in providing mental health care, it did not appear that champions had emerged among the more permanent front-line HIV staff, which could have compensated for a lack of higher level support.

##### Staff rotation policies

The bidirectional relationships promoted by the program were threatened by hospital policies that required most nursing staff to rotate among services at fixed intervals .The rotation system was both a threat to the maintenance of working relationships and a disincentive to learn additional skills perceived of as not being of long-term use.

#### Capacity factors that promoted sustainability

##### Workforce

From its conception, hope for the program's sustainability had been built on the development of working relationships between HIV providers and on-site mental health personnel (mostly mental health nurses who were long-term staff members). The program's presence appeared to have raised the profile of mental health nurses who were already at the sites and allowed them to resume or expand their mental health roles.

#### Capacity factors that hindered sustainability

##### Workforce

While mental health nurses could potentially remain at their posts for extended periods of time, psychiatrists were more likely to leave for other posts, disrupting relationships with HIV providers. Without the ability to make a personal referral, HIV providers had to fall back on routine referral mechanisms and felt that mental health resources were ‘congested’ and unable to meet their needs.

It also did not appear that attention to mental health had become part of orientation of new staff. Two of the ‘not trained’ providers at one of the sites, who had joined the HIV team relatively soon after the final program training, said they were not aware of integration efforts or mental health resources, despite the presence of mental health trained HIV providers in the same unit. One said, ‘You may get surprised. I only know very recently even about the presence of one psychiatrist at the facility. I don't know really whether there are any conditions conducive for mental health management in the facility.’

#### Process factors that promoted sustainability

##### Strengthening working relationships

Though the HIV providers generally had prior knowledge of the mental health resources at their sites, the intervention strengthened these relationships and made them more functional. There was evidence of more two-way interaction – especially around mental health symptoms that could be related to medical problems. Training of mental health workers in ‘provider-initiated testing’ for HIV resulted in more referrals from mental health providers to HIV providers and opportunities for joint work to make a diagnosis.

#### Process factors that hindered sustainability

##### Informal nature of HIV-mental health collaboration

The program had been successful in promoting collaboration and informal consultation among HIV and mental health providers, but collaborative mechanisms (for example, regular joint rounds) were not established as formal, regular processes and thus were more susceptible to fading out as key participants left and outside attention diminished.

##### Absence of a formal screening and documentation process

Within the larger JHU-TSEHAI program, HIV providers also received trainings for detection and management of tuberculosis, nutritional problems, and opportunistic infections common in immunosuppressed patients. These other programs required formal screening procedures and documentation of the procedure and results. Trained providers noted that the mental health program's lack of a formal screening tool or established place for documentation in the standard encounter form harmed its sustainability once management and external attention diminished.

##### Lack of mechanisms for ongoing evaluation and feedback

The lack of standardized screening and required documentation, noted above, contributed to a lack of ongoing evaluation and feedback. Whereas sites and providers received feedback about rates of routine assessment and documentation of patients’ nutritional status or exposure to tuberculosis, mental health assessment and treatment were not part of required documentation nor were they examined centrally as indicators of the overall HIV program's performance.

## Discussion

Co-training generalists and mental health providers, and actively promoting local collaborations, helped sustain the impact of a task-shifting mental health program more than a year after formal support ended. Though differences may have been diminishing over time, compared with generalist providers who had not been trained, trained providers had different attitudes toward mental health and greater sensitivity detecting potential cases. They made different treatment decisions, and showed more awareness of mental health resources.

Program characteristics that providers felt were related to their sustained mental health efforts included having learned effective means to address common patient problems, receiving positive reinforcement from patients, access to written materials that supported mental health work, and enhanced bi-lateral relationships with mental health providers. The inclusion of these characteristics in the program had resulted from a deliberate attempt to design a program that fit the context in which it would be used (Proctor *et al.* 2015). The program was designed collaboratively by a bi-national team (Wissow *et al.* 2014). It sought to extend current practice in familiar ways (Padmanathan & De Silva, 2013), building on existing values related to holistic care, how HIV providers classified patients’ psychosocial problems, and counseling skills familiar to HIV providers (Assefa *et al.* 2009). Co-training of HIV and mental health clinicians helped create familiarity with each other's work and trusting relationships between clinicians in these two different fields (Benzer *et al.* 2012).

Despite this attention to fit, impact of the program was clearly seen as diminishing over time. Key staff involved in informal networks of care left, and were either not replaced or their replacements did not seek out and interact with HIV providers in the same way (and vice versa). Mental health was not included among the metrics used to assess site performance, making it vulnerable to being set aside when there was competition for limited time. Although administrators had been supportive of the program, they had not taken over responsibility for its on-going implementation. Similarly, though the program had been well-received by front-line HIV staff, champions who could sustain it were not cultivated and did not emerge. Materials were not renewed or updated, and new HIV staff did not receive orientation highlighting the mental health resources available. The training itself may have fallen short in its inability to offer means of dealing with some of the social issues faced by patients, especially stigma that caused them to seek care far from home, limiting possibilities for follow-up, and stress related to poverty and unemployment (Heiman & Artiga, 2015).

Many of these limitations fall under what could be called the ‘absorptive capacity’ of the implementation sites (Emmons *et al.* 2012). The program had many elements promoting long-term impact, but sites did not have the ability to make structural changes that would have supported long-term impact. They could or did not institute processes for tracking mental health care, building mental health collaboration into job descriptions or day-to-day structured activities, or adding mental health materials to inventories of tools that would be routinely available and regularly updated.

### Limitations

The quantitative results come from a small, non-randomized cross-sectional study. We can say little about differences or similarities between trained and not-trained providers outside of their professional category and training status. We cannot rule out the possibility that trained providers were simply more experienced or more predisposed to treat mental health problems. We have only post-intervention data so we can only infer from the contrasts seen that the impact on provider behavior is attributable to training, and we cannot provide data demonstrating that the altered provider behavior actually led to better patient outcomes. Our main quantitative evidence for sustained impact was greater agreement with standard screening instruments, and better sensitivity without significant loss of specificity. Studies in other low and middle income countries settings have shown mixed results for changes in case detection after task-shifting training (Goncalves *et al.* 2013; Jenkins *et al.* 2013; Kauye *et al.* 2014). The changes we found were modest but consistent with training content. The majority of the training covered common mental disorders and emphasized the importance of eliciting patients’ concerns. Interview responses suggested that trained providers were more likely to ask about patients’ mood and to see beyond interactions with patients who were initially angry or irritable.

The range of informants we spoke with was also limited. We spoke only to HIV clinicians, and only at two of the seven original implementation sites. Though these sites served the large majority of patients seen in the program, the fact that the other sites were in more remote parts of the country could have led to different degrees of sustained impact and different explanations for it. We might have gained other insights from talking to mental health providers and administrators, and, indeed, our results suggest that these perspectives could be particularly important to verifying our conclusions about how the program could have been more strongly sustained.

## Conclusions

Efforts to fit this task-shifting program to its target context, and promotion of potentially self-sustaining consultation networks across professional lines, were associated with a modest but measureable degree of sustained impact more than a year after formal program support ended. More attention to the capacity of the sites to sustain the program – institutionalizing it with monitoring, personnel processes (reducing staff rotation, including mental health in ‘on-boarding’), and renewal of mental health-related staff support materials might have helped institutionalize the changed behaviors that providers reported. The extent to which the sites lacked these capacities related in part to local and national policies regarding data management, hospital staffing, financing for continuing staff education, and mental health as a core clinical priority. Changes to these policies might not have guaranteed the long-term impact of the program, but combined with changes to the program itself (recruiting local champions, broadening interventions to include social determinants of mental health), they might have better sustained providers’ mental health work after active program support ended.

## Supplementary material

For supplementary material accompanying this paper visit http://dx.doi.org/10.1017/gmh.2017.21. click here to view supplementary material
