# Supplementary material for: Factors promoting and inhibiting sustained impact of a mental health task-shifting program for HIV providers in Ethiopia
Source: Glob Ment Health (Camb). 2017 Dec 4;4:e24. doi: 10.1017/gmh.2017.21 (PMC5719476; doi:10.1017/gmh.2017.21)
Supplement: Supplementary file 1 [file S2054425117000218sup001.docx]

ART provider interview guide

**Introduction**

Thank participant for time – remind that they can stop at any point.

Can speak in Amharic or English.

Remind about confidentiality – will not disclose identity of those interviewed or quote them in any identifiable way. Will edit any notes or transcript to remove anything that might identify them.

Ask for permission to record visit. Say that they can ask to turn off the recorder at any time. Remind that will erase the recording as soon as the interview is transcribed.

**Questions for all providers**

Tell me a little bit about the patients that you usually care for.

Probes can include age, gender, where they are from, ethnicity, range of illnesses and HIV severity.

What would you say are the most common mental health problems among your patients?

Probes can include: don’t worry so much about diagnoses – what kinds of concerns do they have?

Do your patients with mental health problems seek care for them from you?

Probes – where else do you think they seek care?

What sorts of mental health treatment do you most commonly provide?

What other mental health resources are available to your patients?

Probes – do you believe that they visit traditional healers? Get advice from their families? See the mental health providers here?

If you have a question about mental health treatment, is there a way for you to get assistance?

Probes – do you even consult the mental health nurse or another clinician informally?

Are there barriers to getting help for your patients’ mental health problems?

Probes can include: expense, time, no specialists available, no medications available, the patients’ own beliefs.

Would you be interested in any further mental health training?

**Additional questions for trained providers**

Can you give us an example of a patient who had a problem that the training helped you identify, treat, or refer?

Can you give us an example of a patient who had a problem (or a kind of problem) that you still feel you would have difficulty helping?

Were there parts of the training that seemed most helpful?

Was there anything that got in the way of your ability to use the information and skills that you learned in the training?

**Thank provider for participation**
